# Consulted ethical problems of clinical nursing practice: perspective of faculty members in Japan

**DOI:** 10.1186/s12912-017-0217-3

**Published:** 2017-05-12

**Authors:** Mari Tsuruwaka

**Affiliations:** Department of Nursing, St. Luke’s International University, 10-1 Akashi-cho, Chuo-ku, Tokyo 104-0044 Japan

**Keywords:** Ethical problems, Clinical nursing practice, Japanese student nurses, Nursing ethics, Nursing faculty members

## Abstract

**Background:**

There are several studies that have targeted student nurses, but few have clarified the details pertaining to the specific ethical problems in clinical practice with the viewpoint of the nursing faculty. This study was to investigate the ethical problems in clinical practice reported by student nurses to Japanese nursing faculty members for the purpose of improving ethics education in clinical practice.

**Method:**

The subjects comprised 705 nursing faculty members (we sent three questionnaires to one university) who managed clinical practice education at 235 Japanese nursing universities. We performed a simple tabulation of the four items shown in the study design. 1) the details of student nurse consultations regarding ethics in clinical practice (involving the students themselves, nurses, care workers, clinical instructors, and nursing faculty members); 2) the methods of ethics education in clinical practice; 3) the difficulties experienced by the nursing faculty members who received the consultations; and 4) the relationship between clinical practice and lectures on ethics. Furthermore, the analysis was based on the idea of ethical principles, respect for persons, beneficence, and justice.

**Results:**

The response rate was 28% (198 questionnaires). The nursing faculty members were consulted for various problems by student nurses. The details of these consultations were characterized by the principles of respect for patient by nurses, the principles of benevolence by faculty and clinical instructors, and the principle of justice pertaining to evaluations. The results indicate that there is an awareness among the nursing faculty regarding the necessity of some sort of ethics education at clinical settings. Moreover, based on the nature of the contents of the consultations regarding the hospital and staff, it was evident that the nursing faculty struggled in providing responses. More than half of subjects exhibited an awareness of the relationship between the classroom lectures on ethics and clinical practice.

**Conclusion:**

The results suggest the need for analyzing the ethical viewpoints of student nurses, prior learning, and collaboration with related courses as part of ethics education in clinical practice.

**Electronic supplementary material:**

The online version of this article (doi:10.1186/s12912-017-0217-3) contains supplementary material, which is available to authorized users.

## Background

Although ethics is an important topic in nursing education, there has not been much discussion regarding appropriate teaching methods for this topic [1, 2]. “Bioethics” and “Nursing ethics” are regarded as necessary subjects in nursing education, and nursing ethics is a compulsory subject in many Japanese nursing universities [3]. During clinical practice, student nurses learn specific ethical practices such as methods to protect the rights of patients and respecting the dignity of patients through interactions with practicing nurses. The most appropriate way to learn ethics in nursing is through clinical practice.

Many of the ethical problems encountered by student nurses in clinical practice are related to the actions of the nursing staff toward patients, and research on the topic has been conducted in various countries [4–6]. Recent studies from New Zealand, Brazil, and UK have reported that the will of patients is not respected and that patient rights and privacy are not protected [7–9]. In Japan, problems related to the level of respect paid by nurses to patients has primarily been reported [5, 10, 11]. Issues such as not telling patients the truth [12] and the inappropriate provision of information [13] were also indicated as ethical problems. Student nurses who were the subjects of these investigations were typically at least third-year students; however, these students reportedly also experienced various ethical problems during fundamental clinical practice [14]. The results of one study regarding the experience of student nurses during clinical practice revealed that the nursing faculty offered very little support [7]. Moreover, it has been reported that student nurses tend to consult with other student nurses, family members, or friends rather than with the nursing faculty [15, 16]. An investigation of 132 Japanese student nurses revealed that only 19.7% consulted the nursing faculty with respect to ethical problems experienced in clinical practice owing to the fear of negative evaluation [17]. In another study, student nurses who did consult the nursing faculty reportedly obtained emotional support and appropriate information [18].

Ethical problems created by the nursing faculty for student nurses in clinical practice primarily comprise negative feedback in front of others [19–21] and unfair evaluations [20, 22, 23]. It has been indicated that student nurses experience a power gap between themselves and the nursing faculty and attempt to preserve the relationship; [23] similar results have also been identified in Japan [6].

Fowler et al. identified “bullying of student nurses”, “racial discrimination” and “animosity and intolerance” in the clinical practice facility to be the ethical problems experienced during clinical practice education [24]. Bullying or disrespectful remarks made by nurses toward student nurses [7] reportedly cause moral distress to student nurses [25]. This effect was also observed for other specializations, but the problem was the most serious among nurses. In addition, some instructors and nursing faculty memebers reportedly justified this bullying behavior (e.g., looking down upon or taking an intimidating stance) toward student nurses [26]. Moreover, this behavior was not limited to nursing clinical practice but expanded to other types of healthcare clinical practices. Such experiences were found to lead to decreased motivation among students [15].

Although the above studies describe investigations that targeted student nurses, we should now examine the results of studies that investigated nursing faculty members. In Iran, the unethical behaviors of student nurses, as observed by nursing faculty, members included a lack of respect, dishonesty, and self-centeredness [27]. In Japan, surveys of nursing faculty have indicated that the words and behavior of student nurses lack respect toward patients during clinical practice [28–30].

Thus, there are several studies that have targeted student nurses, but few have clarified the details pertaining to the specific ethical problems in clinical practice with the viewpoint of the nursing faculty. Therefore, the present study aimed to elucidate the details of the consultations with Japanese nursing university faculty members by student nurses regarding the various ethical problems faced by the students and to assess the problems in ethics education during clinical practice. The present study was conducted in order to answer the following two questions: 1) Do Japanese nursing faculty receive consultations regarding ethical problems that student nurses encountered during clinical practice? 2) What ethical problems do student nurses bring up when they consult with nursing faculty during their clinical practice?

## Methods

### Study design

The present study used an anonymous self-administered questionnaire to elucidate the following four points: 1) the details of student nurse consultations regarding ethics in clinical practice (involving the students themselves, nurses, care workers, clinical instructors, and university faculty members); 2) the methods of ethics education in clinical practice; 3) the difficulties experienced by the nursing faculty members who received the consultations; and 4) the relationship between clinical practice and lectures on ethics. The questionnaire used in this survey was not a pre-existing questionnaire; it was developed by the researchers themselves (Additional file 1). For point 1) described above, the answer choices were based on the results of a survey that was previously conducted by the researchers regarding ethical problems encountered by Japanese nursing students during clinical practice as well as the Japanese Nursing Association’s Code of Ethics for Nurses. The details are listed below.

The choices for the answers to the question regarding ethical problems related to patients were based on the ethical guidelines of the Japanese Nursing Association (e.g., “respect of the individual,” “respect of intention,” “freedom of behavior,” “consideration of privacy,” “consideration of shame,” “guarantee of the right to know,” “collaboration with other health care workers,” “care that is based on trust,” “responsibility of nursing acts performed,” and “promotion of recovery”). Moreover, based on Tsuruwaka [6], the ethical problems that developed in the educational field were presented as choices for problems that Japanese student nurses often encounter in clinical practice, including “negative feedback in public,” “overly emotional words/behavior and attitude,” “inconsistent instructions among nursing faculty,” “inconsistent instructions among nursing faculty and nursing staff,” “instructions differing from those provided to other student nurses,” “adequate rest not provided,” “forced to perform training record-related tasks to the point of being unable to sleep,” “clinical practice evaluation being unfair,” “remarks regarding obtaining course units,” “lack of consideration of the relationships between student nurses,” and “sharing the personal information of student nurses with others without intimating the students in question.”

For point 2) mentioned above, the questions created by the researchers were designed to inquire about the education required to handle ethical issues related to clinical practice that were the subjects of consultations by student nurses. The seven answer choices were as follows (multiple answers allowed): Instructions to consult with nursing faculty and clinical instructors and obtain advice, making the nursing student consider what type of ethical problem it is, creating an opportunity for the students to think about ethical issues likely to be encountered, making the student think about concrete actions to take, instructions on knowledge in fundamental nursing classes before clinical practice, instructions on knowledge in independent nursing ethics classes before clinical practice, and no special education provided. Regarding point 3), the answer choices were pertaining to the frequency (rated on a 4-step scale) with which nursing faculty members experienced difficulties related to consultations by students. The following nine were difficulties that the subjects answered that they experienced “frequently” and “somewhat.” They comprised difficulty in coping with issues related to the hospital and staff, differences between university education and clinical practice, experiencing a lack of competence, having differences of opinion with other instructors, ending up behaving in way that shows deference to the clinical practice instructors and staff at the clinical practice facility, having no extra time to spare, prioritizing issues to be covered during the clinical practice, not knowing what to think with respect to the problem, and not knowing how to approach student nurses. Point 4) asked to what extent (on a 4-step scale) they perceived the relationship between clinical practice and lectures.

### Subjects

The subjects comprised 705 nursing faculty members who managed clinical practice education at 235 Japanese nursing universities. Nursing faculty were surveyed during the period from March 1, 2015 through March 30 by self-reported questionnaire. We sent three questionnaires to one university and requested that they be distributed to three faculty members who were responsible for leading clinical practice (one of whom was involved in the field of fundamental nursing). In the field of fundamental nursing, the faculty members are often responsible for conducting courses on nursing ethics, and previous studies have shown that student nurses often experience ethical problems in clinical practice [14].

### Ethical considerations

The subjects were provided with an explanation of the purpose, methods, and ethical considerations of the present study, and the voluntariness of the study was ensured. We informed the subjects that the following steps would be taken: 1) questionnaires would be anonymous and could not be used to identify any personal information, 2) returning the questionnaire through mail would be considered to indicate obtainment of consent, 3) The questionnaires would be strictly managed within the locker of the researcher’s office, which itself is always kept locked. 4) the questionnaires would be stored for at least 5 years after publishing the data in an academic journal and subsequently shredded and discarded, and 5) anonymity would be ensured when the data will be published in an academic journal. The present study was approved by the Institutional Review Board of St. Luke’s International University (No. 14–061).

### Data analysis

We performed a simple tabulation of the four items shown in the study design, and our analysis was based on the idea of the three major ethical principles, respect for persons, beneficence, and justice by Theis [21]. Items regarding paying respect to the subject as a human were categorized as principles of respect for persons, items regarding not causing any pain or detriment to the subject and providing appropriate guidance were categorized as principles of beneficence, and items regarding equal treatment of subjects and an appropriate evaluation were categorized as the principles of justice.

## Results

### Basic attributes of subjects

The recovery rate was 28% (198 questionnaires). Table 1 explains characteristics and background of respondents. The subjects were women (94%), men (4%) and unknown (2%). The subjects comprised those in their 30s (18%), 40s (36%), 50s (35%), and 60s (11%). More than 70% of subjects were in their 40s and 50s. The job titles of the subjects were as follows: professor (24%), associate professor (25%), lecturer (21%), and assistant professor (24%). The fields of specialty of the faculty members responsible for clinical practice were as follows: fundamental (34%), adult (28%), geriatric (12%), maternal (8%), pediatric (7%), psychiatric (6%), home (2%), and public health (2%) nursing.

**Table 1:** Characteristics and background of respondents (*n* = 198)

Characteristics and background		Number of respondents	Percentage
Sex	female	186	94%
	male	8	4%
	no response	4	2%
Age-group	30s	36	18%
	40s	72	36%
	50s	69	35%
	60s	21	11%
Job title	professor	48	24%
	associate professor	50	25%
	lecturer	42	21%
	assistant professor	48	24%
	assistant	3	2%
	no response	7	4%
Fields of specialty	fundamental nursing	67	34%
	adult nursing	56	28%
	gerontological nursing	24	12%
	maternity nursing	16	16%
	pediatric nursing	13	7%
	psychiatric nursing	12	6%
	home care nursing	4	2%
	public health nursing	4	2%
	global health nursing	1	1%
	no response	1	1%

### Awareness of nursing faculty regarding student nurses considering ethics in clinical practice

Question regarding the situations in which students experience ethical issues during clinical practice was as follows: “speech/behavior and attitude of the staff toward patients” (84%), “privacy-related issues” (83%), “issues related to medical care and nursing behaviors (the act of medical care/nursing itself that is being undertaken)” (78%), “issues related to care methods (methods of nursing care)” (73%), “issues regarding patient rights” (69%), “immaturity of fundamental nursing skills and lack of self-confidence in student nurses” (45%), “speech/behavior and attitude of clinical instructors toward student nurses” (40%), “speech/behavior and attitude of the nursing faculty toward student nurses” (36%), “clinical practice instructions provided by clinical instructors” (29%), “issues regarding organization and management” (28%), “clinical practices instructions of the nursing faculty” (26%), “medical staff cooperation” (24%), and “assessment of clinical practice” (14%).

### Details of the consultations received by nursing faculty

#### Regarding student nurses

Consultations regarding the behaviors of the students themselves were as follows: “inability to appropriately communicate with patients owing to insufficient knowledge and skills” (70%), “inability to provide appropriate care owing to insufficient knowledge and skills” (57%), “inability to respect the patient’s will” (49%), “inability to protect the patient’s privacy” (35%), “inability to protect the patient’s rights” (16%), “improper posting of occurrences during training on social networking service” (10%), “inability to properly prevent infection” (8%), and “no consultations from by students” (7%).

#### Regarding nurses

Table 2 explains the consultations regarding nurses by student nurses. Consultations regarding nurses were as follows: “speech/behavior that does not respect the personality of the patient” (56%), “inappropriate consideration of shame (54%),” “inappropriate consideration of privacy (47%),” “lack of respect for the wishes of the patient” (45%), “intimidating behavior toward patients” (34%), “limiting the freedom of the actions of the patients” (28%), “treating patients as objects rather than human” (27%), “not guaranteeing the right of the patient to know” (16%), “insufficient cooperation with other departments, causing detriment to the patient” (15%), “causing the patient physical pain” (13%), “not providing care that is based on trust” (11%), “ignoring the patient” (11%), “no consultation with nurses” (9%), “not taking responsibility for the nursing care provided” (5%), and “actions that interfere with the recovery of the patient” (5%).

**Table 2:** Ethical problems regarding nurses and care workers encountered by student nurses (*n* = 198, multiple answers)

	Consultations regarding nurses		Consultations regarding care workers	
Ethical problems	Number of respondents	%	Number of respondents	%
Speech/behavior that does not respect the personality of the patient	110	56	48	24
Inappropriate consideration of shame	107	54	50	25
Inappropriate consideration of privacy	94	47	39	20
Lack of respect for the wishes of the patient	90	45	37	19
Intimidating behavior toward patients	68	34	27	14
Limiting the freedom of the actions of the patients	56	28	27	14
Treating patients as objects rather than human	53	27	32	16
Not guaranteeing the right of the patient to know	31	16	2	1
Insufficient cooperation with other departments, causing detriment to the patient	30	15	7	4
Causing the patient physical pain	25	13	7	4
Not providing care that is based on trust	22	11	6	3
Ignoring the patient	21	11	17	9
Not taking responsibility for the nursing care (or care) provided	9	5	3	2
Actions that interfere with the recovery of the patient	9	5	4	2
No consultation with nurses (or care workers)	18	9	85	43

Based on these results, we elucidated that many of the specific consultations on nurses initiated by the students were related to the respect of patients: “behaviors that do not respect the personality of the patient,” “inappropriate consideration of shame,” “inappropriate consideration of privacy,” and “failure to respect patient wishes,” among others. We hypothesized that students are sensitive to these issues because they often provide routine support to patients, such as care related to personal hygiene in practice.

#### Regarding care workers

Table 2 explains the consultations regarding care workers by student nurses. Consultations regarding care workers were as follows: “inappropriate consideration of shame” (25%), “speech/behavior that does not respect the personality of the patient” (24%), “inappropriate consideration of privacy” (20%), “lack of respect for the wishes of the patient” (19%), “treating the patient as an object” (16%), “intimidating behavior toward the patient” (14%), “limiting the freedom of the patient’s behavior” (14%), “ignoring the patient” (9%), “causing the patient physical pain” (4%), “insufficient cooperation with other departments, causing detriment to the patient” (4%), “not providing care that is based on trust” (3%), “actions that interfere with the recovery of the patient” (2%), “not taking responsibility for the nursing care provided” (2%), and “not guaranteeing the right of the patient to know” (1%). Some clinical practice facilities did not have long-term care workers, and the answer with the highest frequency was “there were no consultations regarding the long-term care workers” (43%). Thus, similar to consultations on nursing, many issues regarding care workers consulted by students included those related to respect of patients, such as “behaviors that do not respect for the patient” and “inappropriate consideration of shame.”

#### Regarding clinical instructors

Table 3 explains consultations regarding clinical instractors by student nurses. Consultations regarding clinical instructors were as follows: “overly emotional speech/behavior and attitude” (53%), “inconsistent instructions among nursing faculty” (43%), “negative feedback in public” (35%), “instructions differing from those provided to other student nurses” (20%), “no consultations with clinical instructors” (14%), “sharing personal information of student nurses with others without intimating the students in question” (5%), “unfair clinical practice evaluation” (5%), “remarks regarding obtaining course units” (3%), “adequate rest not provided” (3%), and “lack of consideration of the relationships among student nurses” (3%).

**Table 3:** Ethical problems regarding nursing faculty and clinical instructors encountered by student nurses (*n* = 198, multiple answers)

	Consultations regarding nursing faculty		Consultations regarding clinical instructors	
Ethical problems	Number of respondents	%	Number of respondents	%
Inconsistent instructions among the nursing faculty members	110	56	85	43
Overly emotional speech/behavior and attitude	78	38	104	53
Inconsistent instructions among the nursing faculty and nursing staff	72	36	-	-
Negative feedback in public	63	32	70	35
Instructions differing from those provided to other student nurses	58	29	39	20
Forced to perform training record-related tasks to the point of being unable to sleep	52	26	-	-
Unfair clinical practice evaluation	36	18	10	5
Remarks regarding obtaining course unit	31	16	6	3
Lack of consideration of the relationships among student nurses	12	6	5	3
Sharing the personal information of student nurses with others without intimating the students in question	11	6	10	5
Adequate rest not provided	7	4	5	3
No consultation with the nursing faculty (or clinical nurse educator)	21	11	27	14

Therefore, student consultations regarding clinical instructors were often related to principles of beneficence, such as “overly emotional speech/behavior and attitude” and “inconsistent instructions among the nursing faculty members.”

### Regarding nursing faculty

Table 3 explains consultations regarding nursing faculty by student nurses. Consultations regarding the nursing faculty were as follows: “inconsistent instructions among the nursing faculty members” (56%), “overly emotional speech/behavior and attitude” (38%), “inconsistent instructions among the nursing faculty and nursing staff” (36%), “negative feedback in public” (32%), “instructions differing from those provided to other student nurses” (29%), “forced to perform training record-related tasks to the point of being unable to sleep” (26%), “unfair clinical practice evaluation” (18%), “remarks regarding obtaining course units” (16%), “no consultation with the nursing faculty” (11%), “lack of consideration of the relationships among student nurses” (6%), “sharing the personal information of student nurses with others without intimating the students in question” (6%), and “adequate rest not provided” (4%).

Therefore, student consultations regarding instructors were often related to the principles of beneficence, such as “inconsistent instructions among nursing faculty members.”

### Specific education method envisioned by nursing faculty

Ethical problems regarding education in clinical practice regarding the consulation with student nurses were as follows: “instructions to consult with nursing faculty and clinical instructors and obtain advice” (72%), “making the nursing student consider what type of ethical problem it is” (64%), “creating an opportunity for the students to think about ethical issues likely to be encountered” (57%), “making the student think about concrete actions to take” (53%), “instructions on knowledge in fundamental nursing classes before clinical practice” (41%), “instructions on knowledge in independent nursing ethics classes before clinical practice” (38%), and “no special education provided” (0%). The above results indicate that there is an awareness among the nursing faculty regarding the necessity of some sort of education. It became evident from the analysis that there was a need to connect the consultation with education to perform analysis based on ethical viewpoints, implement prior learning, and collaborate with relevant courses.

### Concerns of nursing faculty

Regarding the question of whether the nursing faculty members struggled with how to respond to consultations from student nurses on the ethical problems that occurred during clinical practice, the responses were as follows: “frequently” (9%), “somewhat” (45%), “not much” (28%), “no” (11%), and “I have never been consulted” (2%). Thus, “frequently” and “somewhat” comprised 54% of total responses and “not much” and “no” comprised 39%, revealing that many nursing faculty members struggled to some extent with providing responses. Specific difficulties experienced by the 108 subjects who responded “frequently” or “somewhat” were as follows: “difficulty in coping with issues related to the hospital and staff” (48%), “differences between university education and clinical practice” (28%), “experiencing a lack of competence” (20%), “having differences of opinion with other instructors” (15%), “ending up behaving in way that shows deference to the clinical practice instructors and staff at the clinical practice facility” (15%), “having no extra time to spare” (14%), “prioritizing issues to be covered during the clinical practice” (9%), “not knowing what to think with repect to the problem” (4%), and “not knowing how to approach student nurses” (3%). Thus, from the nature of the contents of the consultations regarding the hospital and staff, it was evident that the nursing faculty struggled in providing responses.

### Awareness of the relationship between classroom lectures and ethics

Regarding the relationship with the independent lecture courses of “nursing ethics” and “bioethics,” the responses were as follows: “very aware” (21%), “somewhat aware” (35%), “not very aware” (30%), and “not at all aware” (5%). Thus, more than half the total subjects exhibited an awareness of the relationship between the classroom lectures of ethics and clinical practice.

## Discussion

The results of the present study revealed that Japanese nursing faculty received consultations regarding various ethical problems that student nurses encountered during clinical practice. The details of the consultations regarding specialized professions (e.g., nursing) were characterized by the principle of respect for persons, whereas consultations regarding the nursing faculty and clinical instructors were primarily concerned with beneficence (e.g., providing instructions that were considered inappropriate) and principles related to justice (e.g., fair evaluation of clinical practice). The nursing faculty considered patient-related issues to be the most common ethical problem encountered by student nurses; however, they also received similar consultations regarding problems related to instructions during clinical practice. The details of the consultations revealed by the present study are consistent with those reported in previous studies regarding ethical problems in clinical practice [4–9]. Thus, Japanese student nurses demonstrate some form of interest in ethical problems in clinical practice through consultations with nursing faculty. The consultations should be understood as optimal opportunities for use in education. The subjects of the present study considered three points to be necessary for education: 1) analysis of the ethical viewpoints of student nurses based on experienced phenomena, 2) prior studies, and 3) collaboration with related subjects.

Regarding the awareness of student nurses during clinical practice, it is important to first have the students consider the reasons and background for the discomfort that they feel and encourage them to vocalize the phenomena that they have experienced. It is also necessary to examine the nature and characteristics of these problems using the code of ethics and other ethical principles. Therefore, it is possible to view these phenomena from an ethical viewpoint through this process. Regarding the details of the consultations of student nurses, it may be useful to first spare time to consult with student nurses in addition to facilitating a forum for discussion on universal themes for all students.

It is also important to demonstrate ethical problems that student nurses may encounter to use as opportunities for prior learning. Moreover, it is ideal to obtain the cooperation of student nurses who have completed the clinical training in case creation to incorporate their viewpoints. It may also be beneficial to include senior students in such case studies.

The results of the present study revealed that the nursing faculty was conscious of ethics-related issues in classroom learning. It may also be useful to incorporate the essence into the lecture subjects and deepen the ethical discussion of both sides while exchanging information regarding the ethical issues that students encounter during clinical practice. This can be achieved through conversation with faculty members who are responsible for conducting independent courses.

More than half the subjects were concerned about how to respond when consulted by student nurses regarding ethical problems that arose during clinical practice. Consultations related to the hospital and ethical problems involving patients by staff resulted in a difficult situation for the nursing faculty. The importance of respecting the personality of patients is taught in various lectures. However, there are cases in which student nurses see that this is not respected in clinical practice. It may be easier to provide instructions that deepen the learning of students, as observed in the practice model. However, when they become aware of inappropriate attitude and care by nurses, how should faculty members respond and what kind of education is appropriate? An investigation of nursing faculty members in Africa [31] revealed that student nurses are afraid to vocalize criticism regarding nursing practice and that there is a lack of critical discussion and reflection to enhance moral capacity. When student nurses comment on the inappropriate care of patients by nurses, the nurses think negatively of them, and the authoritarian learning environment experienced by student nurses reportedly influences their remarks and motivation.

First, it is desirable for nursing faculty not to negate the awareness of student nurses in clinical practice. In addition, it is important to provide education that allows students to compare their experiences with the lecture content they receive and determine the reason why the students felt uncomfortable about situations that occurred in clinical practice. Paying attention to the creation of relationships between the clinical staff and student nurses on a daily basis and creating opportunities for frank discussion during and after clinical practice may be effective.

The findings are that consultations related to the hospital and ethical problems involving patients by nurses resulted in a difficult situation for the nursing faculty. Regarding the inappropriate attitude and care of patients by the nurses, because the nursing faculty feel that these problems should be dealt with internally by the hospital, they often miss opportunities to discuss important ethical issues with students. When student nurses experience ethical issues it is possible to deepen their thought and reflect and develop further knowledge and skills through appropriate support [32]. If issues (e.g., the character of the patients not being respected) repeatedly occur in clinical practice, it is necessary for the faculty to make an effort to share and review information with other nursing faculty members. Moreover, further discussion with clinical instructors and administrators is necessary to protect the rights of the patients. Through this method, a better environment for clinical practice can be created.

The results of the present study indicate that it is important for nursing faculty to provide instructions that promote consultations by nursing students. However, the provision of instructions alone will not facilitate student consultations. This is because student nurses are always concerned about the effects of such actions on their evaluation. Even when students had doubts about the grades they received, they encountered difficulties and various barriers in communicating these doubts to the nursing educator. For instance, students felt that they might harm their relationship with the faculty or that there was a power differential between themselves and nursing educators [17]. Doi et al. conducted a study of 132 nursing students showing that, due to this fear, only 19.7% consulted with nursing educators when they had ethical concerns about clinical practice [23]. These factors likely came into play in the present study as well, as seen in the fact that consultations about the evaluation students received. The importance of the relationship between student nurses and clinical staff [33, 34] and between the nursing faculty and student nurses [35] in nursing clinical practice education has been indicated, and good relationships are said to promote clinical learning [36]. The important of good relationships with student nurses are as follows: 1) student nurses are inspired and treated as learners, team members, and individuals [37] and 2) mutual respect and openness is directed towards them [38]. This should lead to the creation of an environment in which student nurses can consult without undue worries. Due to the low response rate in this study, care should be taken when attempting to generalize the results.

## Conclusions

The results of this study demonstrated that Japanese nursing instructors received consultations from nursing students related to various ethical problems that students encounter during clinical practice. The details of the consultations were characterized by problems related to words/behavior of nursing instructors that did not respect individual patient personalities, inconsistent instructions among the nursing faculty members and clinical instructors, and overly emotional instructions Details of the ethical problems encountered by student nurses during clinical practice accurately reflect the ethics of nursing practice and ethicality of nurses. Therefore, consultations by student nurses demonstrate optimal opportunities for the students to think independently about nursing and grow as a result. However, there is also a need for the nursing faculty to recognize these opportunities and actively consider nursing practice ethics in their interactions with student nurses. A large-scale study of these issues including nursing schools needs to be conducted in the future. There is also a need to elucidate how instructors, clinical staff members, and students view the ethical problems perceived by students during clinical practice by using qualitative research techniques, such as interview surveys, investigating approaches to ethics education to be taken in future clinical practices.

## Additional file

Additional file 1: Questionnaire English version, The Survey of Ethics Education in Nursing Practice. (PDF 266 kb)
