# Supplementary material for: Consulted ethical problems of clinical nursing practice: perspective of faculty members in Japan
Source: BMC Nurs. 2017 May 12;16:23. doi: 10.1186/s12912-017-0217-3 (PMC5427562; doi:10.1186/s12912-017-0217-3)
Supplement: Additional file 1: — Questionnaire English version, The Survey of Ethics Education in Nursing Practice. (PDF 266 kb) [file 12912_2017_217_MOESM1_ESM.pdf]

The Survey of Ethics Education in Nursing Practice

Please check the corresponding boxes and complete the sections requiring written answers.

Ⅰ Questions related to yourself and the university or college where you work.

（1） sex ☐ male ☐ female

（2） age ☐ 20's ☐ 30's ☐ 40's ☐ 50's ☐ 60's ☐ 70's

（3） job position ☐ professor ☐ associate professor ☐ lecturer ☐ assistant professor

（4） When did you receive the basic nursing education?

☐ 1950's ☐ 1960's ☐ 1970's ☐ 1980's ☐ other（ ）

（5） Please fill out years of experience in teaching by the end of December 2014

【 】year 【 】month

（6） What is your field of specialty in nursing practice ?

（7） What subjects do you take charge of related to nursing ethics or ethics in the university where you work?

☐ nursing ethics class offered as an independent course
☐ nursing ethics class in the area of fundamental nursing
☐ bioethics
☐ medical ethics
☐ ethics
☐ ethics in each area of the nursing profession (eg. ethical problems in maternity nursing, etc.）
☐ other（ ）
☐ not applicable

（8） Please check the box about the university or college where you work.

Type of universities ☐ university ☐ college
Managing organization ☐ national government ☐ prefectures ☐ educational corporations

Ⅱ Questions related to ethics education in nursing practice

（1） Please fill out years of experience in teaching of nursing practice in a university where you worked

【 】year 【 】month （by the end of December 2014）

（2） What do you explain to student nurses in the orientation before nursing practice（multiple answers allowed）

☐ responsibility of student nurses in nursing practice
☐ expected manner and attitude of student nurses
☐ ethical considerations in using social networking service
☐ protection of patients' information in nursing practice
☐ privacy protection
☐ how to handle the writing practice reports based on protection of personal information
☐ violence and harassment
☐ prevention of infection
☐ prevention of medial accidents
☐ response in case of accident
☐ other(   )

（3） In what kind of situations do student nurses think about ethical problems in nursing practice in your teaching experience?（multiple answers allowed）

☐ immaturity of fundamental nursing skills and lack of self-confidence in student nurses
☐ speech/behavior and attitude of the staff toward patients
☐ issues related to care methods (methods of nursing care) ☐ issues regarding patient rights
☐ privacy-related issues ☐ medical staff cooperation
☐ issues related to medical care and nursing behaviors
☐ clinical practice instructions provided by clinical instructors
☐ speech/behavior and attitude of clinical instructors toward student nurses
☐ clinical practices instructions of the nursing faculty
☐ speech/behavior and attitude of the nursing faculty toward student nurses ☐ assessment of clinical practice
☐ issues regarding organization and management
☐ other(   )

（4） Please provide details of the consultations with student nurses regarding ethics in nursing practice
（multiple answers allowed）

【Regarding Student Nurses】

☐ inability to provide appropriate care owing to insufficient knowledge and skills
☐ inability to appropriately communicate with patients owing to insufficient knowledge and skills
☐ inability to respect the patient's will
☐ inability to protect the patient's rights
☐ inability to protect the patient's privacy
☐ improper posting of occurrences during training on social networking service
☐ inability to properly prevent infection
☐ no consultations from by students
☐ other(   )

【 Regarding Nurses】

☐ speech/behavior that does not respect the personality of the patient
☐ treating patients as objects rather than human
☐ intimidating behavior toward patients ☐ inappropriate consideration of shame
☐ lack of respect for the wishes of the patient ☐ ignoring the patient
☐ limiting the freedom of the actions of the patients
☐ causing the patient physical pain ☐ inappropriate consideration of privacy
☐ insufficient cooperation with other departments, causing detriment to the patient
☐ not guaranteeing the right of the patient to know
☐ not taking responsibility for the nursing care provided
☐ not providing care that is based on trust
☐ actions that interfere with the recovery of the patient
☐ no consultation with nurses
☐ other( )

【 Regarding Care Workers】

☐ speech/behavior that does not respect the personality of the patient
☐ treating patients as objects rather than human
☐ intimidating behavior toward patients ☐ inappropriate consideration of shame
☐ lack of respect for the wishes of the patient ☐ ignoring the patient
☐ limiting the freedom of the actions of the patients ☐ causing the patient physical pain
☐ inappropriate consideration of privacy
☐ insufficient cooperation with other departments, causing detriment to the patient
☐ not guaranteeing the right of the patient to know
☐ not taking responsibility for the nursing care provided
☐ not providing care that is based on trust ☐ actions that interfere with the recovery of the patient
☐ no consultations regarding the long-term care workers
☐ other( )

【 Regarding Clinical Instructors】

☐ negative feedback in public ☐ overly emotional words/behavior and attitude
☐ unfair clinical practice evaluation ☐ instructions differing from those provided to other student nurses
☐ adequate rest not provided ☐ inconsistent instructions among nursing faculty
☐ remarks regarding obtaining course units
☐ lack of consideration of the relationships among student nurses
☐ sharing personal information of student nurses with others without intimating the students in question
☐ no consultations with clinical instructors
☐ other( )

【 Regarding Nursing Faculty】

☐ negative feedback in public ☐ overly emotional speech/behavior and attitude
☐ unfair clinical practice evaluation ☐ instructions differing from those provided to other student nurses
☐ adequate rest not provided ☐ inconsistent instructions among the nursing faculty members
☐ remarks regarding obtaining course units
☐ lack of consideration of the relationships among student nurses
☐ inconsistent instructions among the nursing faculty and nursing staff
☐ forced to perform training record-related tasks to the point of being unable to sleep
☐ sharing the personal information of student nurses with others without intimating the students in question
☐ consultation with the nursing faculty
☐ other( )

【Regarding patients】
☐ receiving a present from patients or families
☐ requesting personal contact information by patients or families
☐ suffering violence by patients or families ☐ suffering verbal abuse by patients or families
☐ suffering sexual harassment by patients or families ☐ being ignored by patients or families
☐ other( )

【other】
☐ regarding physicians
( )
☐ regarding other student nurses
( )

Ⅲ（1）When is the optimum time or place to educate student nurses when they encounter ethical problems in nursing practice?（multiple answers allowed）

☐ during nursing practice
☐ after nursing practice, at a reflection meeting, on campus
☐ have an individual interview on campus
☐ create an opportunity to have a discussion with a clinical educator and nursing staff
☐ after clinical practice, during nursing ethics class
☐ no special education required
☐ other( )

（2）What do you think is important for education when student nurses encounter ethical problems during nursing practice?（multiple answers allowed）

- ☐ instructions to consult with nursing faculty and clinical instructors and obtain advice
- ☐ making the nursing student consider what type of ethical problem it is
- ☐ making the student think about concrete actions to take
- ☐ instructions on knowledge in independent nursing ethics classes before clinical practice
- ☐ instructions on knowledge in fundamental nursing classes before clinical practice
- ☐ creating an opportunity for the students to think about ethical issues likely to be encountered
- ☐ no special education provided
- ☐ other(   )

（3）Did you struggle with how to respond to consultations from student nurses regarding the ethical problems that occurred during clinical practice?

☐ frequently ☐ somewhat ☐ not much ☐ no ☐ I have never been consulted

(frequently, somewhat → (4); not much, no, I have never been consulted → Ⅳへ)

（4）What were your specific concerns（multiple answers allowed） Ⅳへ

- ☐ having no extra time to spare
- ☐ not knowing what to think with repect to the problem
- ☐ experiencing a lack of competence
- ☐ difficulty in coping with issues related to the hospital and staff
- ☐ having differences of opinion with other instructors
- ☐ differences between university education and clinical practice
- ☐ not knowing how to approach student nurses
- ☐ prioritizing issues to be covered during the clinical practice
- ☐ ending up behaving in way that shows deference to the clinical practice instructors and staff at the clinical practice facility
- ☐ other(   )

Ⅳ Are you aware of the relationship between classroom lectures and ethics when student nurses encounter the ethical problems in nursing practice.

☐ very aware ☐ somewhat aware ☐ not very aware ☐ not at all aware

Ⅵ Please comment on ethics education in nursing practice.

Thank you very much for your corporation

Please return this questionnaire by the end of March 2015.
